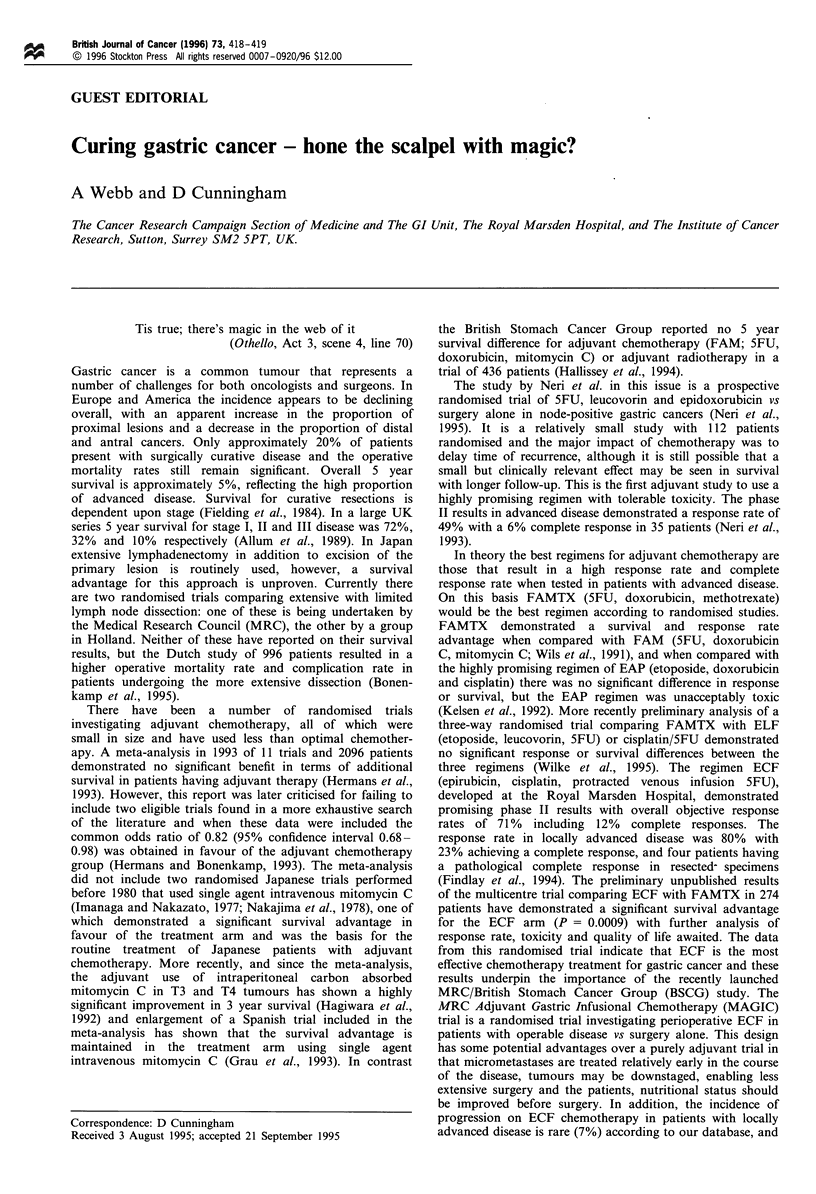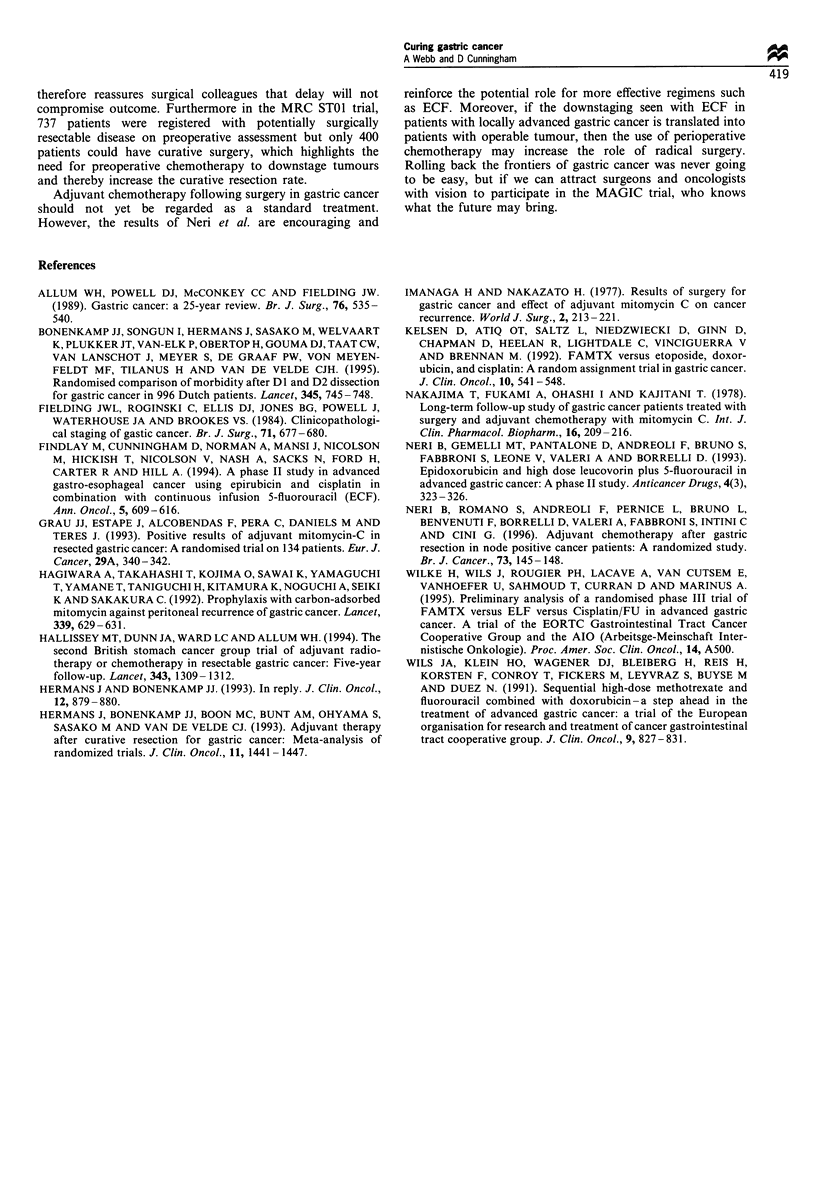# Curing gastric cancer--hone the scalpel with magic?

**DOI:** 10.1038/bjc.1996.74

**Published:** 1996-02

**Authors:** A. Webb, D. Cunningham


British Journal of Cancer (1996) 73, 418-419
© 1996 Stockton Press All rights reserved 0007-0920/96 $12.00

**GUEST EDITORIAL**

### Curing gastric cancer – hone the scalpel with magic?

A Webb and D Cunningham

*The Cancer Research Campaign Section of Medicine and The GI Unit, The Royal Marsden Hospital, and The Institute of Cancer Research, Sutton, Surrey SM2 5PT, UK.*

Tis true; there's magic in the web of it
(*Othello*, Act 3, scene 4, line 70)

Gastric cancer is a common tumour that represents a number of challenges for both oncologists and surgeons. In Europe and America the incidence appears to be declining overall, with an apparent increase in the proportion of proximal lesions and a decrease in the proportion of distal and antral cancers. Only approximately 20% of patients present with surgically curative disease and the operative mortality rates still remain significant. Overall 5 year survival is approximately 5%, reflecting the high proportion of advanced disease. Survival for curative resections is dependent upon stage (Fielding *et al*., 1984). In a large UK series 5 year survival for stage I, II and III disease was 72%, 32% and 10% respectively (Allum *et al*., 1989). In Japan extensive lymphadenectomy in addition to excision of the primary lesion is routinely used, however, a survival advantage for this approach is unproven. Currently there are two randomised trials comparing extensive with limited lymph node dissection: one of these is being undertaken by the Medical Research Council (MRC), the other by a group in Holland. Neither of these have reported on their survival results, but the Dutch study of 996 patients resulted in a higher operative mortality rate and complication rate in patients undergoing the more extensive dissection (Bonenkamp *et al*., 1995).

There have been a number of randomised trials investigating adjuvant chemotherapy, all of which were small in size and have used less than optimal chemotherapy. A meta-analysis in 1993 of 11 trials and 2096 patients demonstrated no significant benefit in terms of additional survival in patients having adjuvant therapy (Hermans *et al*., 1993). However, this report was later criticised for failing to include two eligible trials found in a more exhaustive search of the literature and when these data were included the common odds ratio of 0.82 (95% confidence interval 0.68–0.98) was obtained in favour of the adjuvant chemotherapy group (Hermans and Bonenkamp, 1993). The meta-analysis did not include two randomised Japanese trials performed before 1980 that used single agent intravenous mitomycin C (Imanaga and Nakazato, 1977; Nakajima *et al*., 1978), one of which demonstrated a significant survival advantage in favour of the treatment arm and was the basis for the routine treatment of Japanese patients with adjuvant chemotherapy. More recently, and since the meta-analysis, the adjuvant use of intraperitoneal carbon absorbed mitomycin C in T3 and T4 tumours has shown a highly significant improvement in 3 year survival (Hagiwara *et al*., 1992) and enlargement of a Spanish trial included in the meta-analysis has shown that the survival advantage is maintained in the treatment arm using single agent intravenous mitomycin C (Grau *et al*., 1993). In contrast the British Stomach Cancer Group reported no 5 year survival difference for adjuvant chemotherapy (FAM; 5FU, doxorubicin, mitomycin C) or adjuvant radiotherapy in a trial of 436 patients (Hallissey *et al*., 1994).

The study by Neri *et al*. in this issue is a prospective randomised trial of 5FU, leucovorin and epidoxorubicin *vs* surgery alone in node-positive gastric cancers (Neri *et al*., 1995). It is a relatively small study with 112 patients randomised and the major impact of chemotherapy was to delay time of recurrence, although it is still possible that a small but clinically relevant effect may be seen in survival with longer follow-up. This is the first adjuvant study to use a highly promising regimen with tolerable toxicity. The phase II results in advanced disease demonstrated a response rate of 49% with a 6% complete response in 35 patients (Neri *et al*., 1993).

In theory the best regimens for adjuvant chemotherapy are those that result in a high response rate and complete response rate when tested in patients with advanced disease. On this basis FAMTX (5FU, doxorubicin, methotrexate) would be the best regimen according to randomised studies. FAMTX demonstrated a survival and response rate advantage when compared with FAM (5FU, doxorubicin C, mitomycin C; Wils *et al*., 1991), and when compared with the highly promising regimen of EAP (etoposide, doxorubicin and cisplatin) there was no significant difference in response or survival, but the EAP regimen was unacceptably toxic (Kelsen *et al*., 1992). More recently preliminary analysis of a three-way randomised trial comparing FAMTX with ELF (etoposide, leucovorin, 5FU) or cisplatin/5FU demonstrated no significant response or survival differences between the three regimens (Wilke *et al*., 1995). The regimen ECF (epirubicin, cisplatin, protracted venous infusion 5FU), developed at the Royal Marsden Hospital, demonstrated promising phase II results with overall objective response rates of 71% including 12% complete responses. The response rate in locally advanced disease was 80% with 23% achieving a complete response, and four patients having a pathological complete response in resected specimens (Findlay *et al*., 1994). The preliminary unpublished results of the multicentre trial comparing ECF with FAMTX in 274 patients have demonstrated a significant survival advantage for the ECF arm ($P = 0.0009$) with further analysis of response rate, toxicity and quality of life awaited. The data from this randomised trial indicate that ECF is the most effective chemotherapy treatment for gastric cancer and these results underpin the importance of the recently launched MRC/British Stomach Cancer Group (BSCG) study. The *M*RC *A*djuvant *G*astric *I*nfusional *C*hemotherapy (MAGIC) trial is a randomised trial investigating perioperative ECF in patients with operable disease *vs* surgery alone. This design has some potential advantages over a purely adjuvant trial in that micrometastases are treated relatively early in the course of the disease, tumours may be downstaged, enabling less extensive surgery and the patients, nutritional status should be improved before surgery. In addition, the incidence of progression on ECF chemotherapy in patients with locally advanced disease is rare (7%) according to our database, and

Correspondence: D Cunningham
Received 3 August 1995; accepted 21 September 1995

therefore reassures surgical colleagues that delay will not compromise outcome. Furthermore in the MRC ST01 trial, 737 patients were registered with potentially surgically resectable disease on preoperative assessment but only 400 patients could have curative surgery, which highlights the need for preoperative chemotherapy to downstage tumours and thereby increase the curative resection rate.

Adjuvant chemotherapy following surgery in gastric cancer should not yet be regarded as a standard treatment. However, the results of Neri *et al.* are encouraging and reinforce the potential role for more effective regimens such as ECF. Moreover, if the downstaging seen with ECF in patients with locally advanced gastric cancer is translated into patients with operable tumour, then the use of perioperative chemotherapy may increase the role of radical surgery. Rolling back the frontiers of gastric cancer was never going to be easy, but if we can attract surgeons and oncologists with vision to participate in the MAGIC trial, who knows what the future may bring.

### References

ALLUM WH, POWELL DJ, McCONKEY CC AND FIELDING JW. (1989). Gastric cancer: a 25-year review. *Br. J. Surg.*, **76**, 535-540.

BONENKAMP JJ, SONGUN I, HERMANS J, SASAKO M, WELVAART K, PLUKKER JT, VAN-ELK P, OBERTOP H, GOUMA DJ, TAAT CW, VAN LANSCHOT J, MEYER S, DE GRAAF PW, VON MEYENFELDT MF, TILANUS H AND VAN DE VELDE CJH. (1995). Randomised comparison of morbidity after D1 and D2 dissection for gastric cancer in 996 Dutch patients. *Lancet*, **345**, 745-748.

FIELDING JWL, ROGINSKI C, ELLIS DJ, JONES BG, POWELL J, WATERHOUSE JA AND BROOKES VS. (1984). Clinicopathological staging of gastic cancer. *Br. J. Surg.*, **71**, 677-680.

FINDLAY M, CUNNINGHAM D, NORMAN A, MANSI J, NICOLSON M, HICKISH T, NICOLSON V, NASH A, SACKS N, FORD H, CARTER R AND HILL A. (1994). A phase II study in advanced gastro-esophageal cancer using epirubicin and cisplatin in combination with continuous infusion 5-fluorouracil (ECF). *Ann. Oncol.*, **5**, 609-616.

GRAU JJ, ESTAPE J, ALCOBENDAS F, PERA C, DANIELS M AND TERES J. (1993). Positive results of adjuvant mitomycin-C in resected gastric cancer: A randomised trial on 134 patients. *Eur. J. Cancer*, **29A**, 340-342.

HAGIWARA A, TAKAHASHI T, KOJIMA O, SAWAI K, YAMAGUCHI T, YAMANE T, TANIGUCHI H, KITAMURA K, NOGUCHI A, SEIKI K AND SAKAKURA C. (1992). Prophylaxis with carbon-adsorbed mitomycin against peritoneal recurrence of gastric cancer. *Lancet*, **339**, 629-631.

HALLISSEY MT, DUNN JA, WARD LC AND ALLUM WH. (1994). The second British stomach cancer group trial of adjuvant radiotherapy or chemotherapy in resectable gastric cancer: Five-year follow-up. *Lancet*, **343**, 1309-1312.

HERMANS J AND BONENKAMP JJ. (1993). In reply. *J. Clin. Oncol.*, **12**, 879-880.

HERMANS J, BONENKAMP JJ, BOON MC, BUNT AM, OHYAMA S, SASAKO M AND VAN DE VELDE CJ. (1993). Adjuvant therapy after curative resection for gastric cancer: Meta-analysis of randomized trials. *J. Clin. Oncol.*, **11**, 1441-1447.

IMANAGA H AND NAKAZATO H. (1977). Results of surgery for gastric cancer and effect of adjuvant mitomycin C on cancer recurrence. *World J. Surg.*, **2**, 213-221.

KELSEN D, ATIQ OT, SALTZ L, NIEDZWIECKI D, GINN D, CHAPMAN D, HEELAN R, LIGHTDALE C, VINCIGUERRA V AND BRENNAN M. (1992). FAMTX versus etoposide, doxorubicin, and cisplatin: A random assignment trial in gastric cancer. *J. Clin. Oncol.*, **10**, 541-548.

NAKAJIMA T, FUKAMI A, OHASHI I AND KAJITANI T. (1978). Long-term follow-up study of gastric cancer patients treated with surgery and adjuvant chemotherapy with mitomycin C. *Int. J. Clin. Pharmacol. Biopharm.*, **16**, 209-216.

NERI B, GEMELLI MT, PANTALONE D, ANDREOLI F, BRUNO S, FABBRONI S, LEONE V, VALERI A AND BORRELLI D. (1993). Epidoxorubicin and high dose leucovorin plus 5-fluorouracil in advanced gastric cancer: A phase II study. *Anticancer Drugs*, **4**(3), 323-326.

NERI B, ROMANO S, ANDREOLI F, PERNICE L, BRUNO L, BENVENUTI F, BORRELLI D, VALERI A, FABBRONI S, INTINI C AND CINI G. (1996). Adjuvant chemotherapy after gastric resection in node positive cancer patients: A randomized study. *Br. J. Cancer.*, **73**, 145-148.

WILKE H, WILS J, ROUGIER PH, LACAVE A, VAN CUTSEM E, VANHOEFER U, SAHMOUD T, CURRAN D AND MARINUS A. (1995). Preliminary analysis of a randomised phase III trial of FAMTX versus ELF versus Cisplatin/FU in advanced gastric cancer. A trial of the EORTC Gastrointestinal Tract Cancer Cooperative Group and the AIO (Arbeitsge-Meinschaft Internistische Onkologie). *Proc. Amer. Soc. Clin. Oncol.*, **14**, A500.

WILS JA, KLEIN HO, WAGENER DJ, BLEIBERG H, REIS H, KORSTEN F, CONROY T, FICKERS M, LEYVRAZ S, BUYSE M AND DUEZ N. (1991). Sequential high-dose methotrexate and fluorouracil combined with doxorubicin-a step ahead in the treatment of advanced gastric cancer: a trial of the European organisation for research and treatment of cancer gastrointestinal tract cooperative group. *J. Clin. Oncol.*, **9**, 827-831.